# Dynamics of Phenotypic Heterogeneity Associated with EMT and Stemness during Cancer Progression

**DOI:** 10.3390/jcm8101542

**Published:** 2019-09-25

**Authors:** Mohit Kumar Jolly, Toni Celià-Terrassa

**Affiliations:** 1 Centre for BioSystems Science and Engineering, Indian Institute of Science, Bangalore 560012, India; 2 Cancer Research Program, IMIM (Hospital del Mar Medical Research Institute), 08003 Barcelona, Spain

**Keywords:** cellular dynamics, epithelial-to-mesenchymal transition, cell plasticity, cancer stem cells, mathematical modeling, population homeostasis

## Abstract

Genetic and phenotypic heterogeneity contribute to the generation of diverse tumor cell populations, thus enhancing cancer aggressiveness and therapy resistance. Compared to genetic heterogeneity, a consequence of mutational events, phenotypic heterogeneity arises from dynamic, reversible cell state transitions in response to varying intracellular/extracellular signals. Such phenotypic plasticity enables rapid adaptive responses to various stressful conditions and can have a strong impact on cancer progression. Herein, we have reviewed relevant literature on mechanisms associated with dynamic phenotypic changes and cellular plasticity, such as epithelial–mesenchymal transition (EMT) and cancer stemness, which have been reported to facilitate cancer metastasis. We also discuss how non-cell-autonomous mechanisms such as cell–cell communication can lead to an emergent population-level response in tumors. The molecular mechanisms underlying the complexity of tumor systems are crucial for comprehending cancer progression, and may provide new avenues for designing therapeutic strategies.

## 1. Introduction

Genetic and phenotypic tumor heterogeneity can act as a major bottleneck for the clinical management of cancers [1]. Genetic heterogeneity has been a long-standing focus in cancer progression research [2]. However, non-genetic factors such as phenotypic plasticity [3,4,5] and collective effects resulting from cell–cell communication [6,7,8,9] have gained recent attention for their proposed roles in tumor aggressiveness. Two major interconnected axes of phenotypic plasticity that have been extensively studied across multiple carcinomas are the epithelial–mesenchymal transition (EMT) and cancer stem cell (CSC) plasticity [10,11,12,13,14]. Initially, EMT was hypothesized to be an irreversible event similar to oncogenic transformation and was referred as “epithelial–mesenchymal transformation” [15]. However, during the last decade many studies have demonstrated beyond doubt its dynamic reversible nature in cancer. “Epithelial–mesenchymal plasticity” (EMP) has recently become commonly used terminology, encompassing bidirectional transitions among epithelial (E), mesenchymal (M), and one or more hybrid E/M phenotypes [16]. EMP is a “motor of cellular plasticity” [17], as it accompanies cell changes in immune response [18,19], tumor-initiation potential [8,20,21,22], metabolic reprogramming [23,24], senescence [25], cell proliferation [26,27], and drug resistance [14,28]. Similarly, the “cancer stem cell (CSC) model” initially portrayed CSCs as a small, fixed population which emerge from tissue-specific stem cells at the apex of hierarchical cellular differentiation in tumors. However, recent findings have demonstated the transitionary nature of CSC populations and their different origins from differentiated cell types [29,30]. Thus, EMP and stemness can give rise to dynamic phenotypic heterogeneity in tumors by virtue of their reversibility and plasticity.

Various technological advancements and interdisciplinary cross-fertilization of ideas have led us through these paradigm shifts and emphasized the importance of unraveling the operating principles of cell state transitions along the axes of EMP and/or CSCs. Herein, we have reviewed how investigations at a single-cell level through reporter cell lines, real-time imaging, flow/mass cytometry, high-throughput dynamic measurements—integrated iteratively with mechanism-based mathematical modeling and data-based statistical modeling—have revealed unprecedented insights into the emergent dynamics of cancer progression, at both an intracellular and cell population level.

## 2. Dynamics of EMT

EMT is a nonlinear and reversible trans-differentiation process of an epithelial cell into a mesenchymal phenotype, encompassing changes in multiple phenotypic characteristics such as apico-basal polarity, cell–cell adhesion, cytoskeleton remodeling, cell–matrix adhesion, and cell migration and invasion [31,32]. EMT-inducing transcription factors include ZEB1/2, SNAI1/2, and TWIST, among others. The loss of epithelial molecules such as E-cadherin and the gain of mesenchymal markers such as vimentin and alpha smooth muscle actin (αSMA) represent typical molecular features of EMT [16]. Furthermore, EMT is critical for embryonic development and wound healing, and is involved in pathological conditions such as cancer [16,33]. In cancer progression, EMT has been associated with metastasis, drug-resistance, immune evasion, and reduced patient survival/poor prognosis [14,17,34]. While the dynamics of EMT and its reverse mesenchymal-to-epithelial transition (MET) have been studied in developmental contexts for a long time [16,35], they have only recently received attention in the field of cancer [36,37,38,39,40]. 

EMT and MET have been canonically thought of as “all-or-none” responses, typically because only a few markers were used as a readout at the start and end points of the transition, with little attention to the dynamics and intermediate states. Recently, advanced live-cell imaging [41,42], transcriptomic profiling at multiple timepoints during EMT and/or MET [43,44], flow cytometry [45,46,47], high-throughput single-cell RNA-seq [48,49,50], morphological quantification [51,52], and mass cytometry analysis [53], coupled with mechanism-based mathematical modeling of EMT networks [54], have been used to reveal insights into the dynamics and intermediate states of EMT/MET. While these new sophisticated experimental tools and measurements allow the dynamics of EMT/MET to be tracked in multiple cells using a cohort of markers, mathematical models offer a framework in which to elucidate the mechanisms underlying these dynamics and generate hypotheses that can be experimentally tested. Thus, mathematical models can help to interpret experimental data, unveil complex dynamic patterns, predict cellular responses, and eventually contribute to the design of further expeirments [55]. Remarkably, mathematical models have decongested the understanding of EMT by predicting the existence of stable intermediate EMT or hybrid epithelial/mesenchymal (E/M) states [56,57,58]. Cells in these hybrid E/M phenotypes have been identified in cell lines in vitro and in vivo in primary tumors, circulating tumor cells, and metastases across multiple cancers [46,59,60,61,62]. These hybrid E/M phenotypes may be maintained by “phenotypic stability factors” such as NUMB, OVOL2, GRHL2, and NRF2 [57,63,64,65], a combination of EMT- and MET-inducing signals such as TGF-β and all-trans retinoic acid (ATRA) [66,67], or via cell–cell communication through mechanisms such as Notch–Jagged signaling [68]. Strong evidence for the functional implications of these hybrid E/M phenotype(s) has been reported in both preclinical and clinical settings [46,69]. Examples include (a) their role in tumor formation in mice [20,60], (b) mediating collective cell migration and invasion through aggregates or clusters of circulating tumor cells (CTCs) [70], and (c) the correlation of hybrid E/M signatures with poor patient prognosis in many cancers [71].

Further, these mathematical models have also predicted the co-existence of multiple phenotypes in an otherwise genetically identical population [56]. Such non-genetic heterogeneity has been observed in multiple cell lines, wherein cells harboring both epithelial and mesenchymal signatures were found to co-exist alongside populations predominantly expressing either epithelial or mesenchymal markers [45,46,72]. The relative frequency of these phenotypes can vary depending on the genetic background and other factors, such as the micro-envrionmental milieu or markers used for identification [47,73,74,75]. Nonetheless, the co-existence of different cell subpopulations may enable cooperation among them during metastatic progression [8,9,76,77]. For instance, in vitro and in vivo mixing of more epithelial (PC-3/Mc) and more mesenchymal (PC-3/S) subpopulations of prostate cancer cells was reported to enhance local invasive potential and metastastic colonization of the former [8]. Other studies have documented the influence of paracrine signals from EMT-like cells on non-metastatic cell populations via activation of Hedgehog/GLI signaling to facilitate metastasis [9]. While the exact molecular mechanisms and emergent outcomes of such co-operation are yet to be experimentally determined, these processes are reminiscent of survival strategies observed in diverse ecological systems, such as quorum sensing in bacterial colonies, division of labor, and bet-hedging [78].

**Figure 1:**
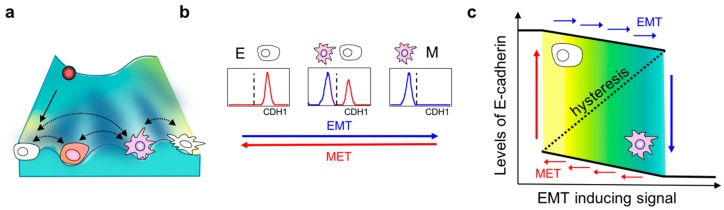
Non-genetic heterogeneity and hysteresis during epithelial–mesenchymal transition (EMT). (**a**) Representation of a Waddington’s landscape with attractors of different EMT states. (**b**) Epithelial cells (left panel) from an isogenic population may respond differently to the same dose of EMT-inducing signals such as TGF-β (middle panel), while all of them may undergo a complete EMT at a higher dose of the signal (right panel). (**c**) Asymmetry in the “forward reaction” and “backward reaction”, i.e., the concentration of the EMT-inducing signal at which all cells switch from being epithelial to mesenchymal (downward blue arrow) is not the same as the one at which all cells switch in the other direction (upward red arrow).

Intriguingly, the co-existence of these distinct phenotypes can be explained by the presence of multiple “attractors” or stable states in the multi-dimensional landscape of epithelial–mesenchymal plasticity. An attractor represents a stable cell phenotype which cells starting with varying levels of molecules can converge towards, depending on the crosstalk among different nodes of an interaction network. The concept of attractors is borrowed from a Waddington’s landscape which depicts how a stem cell progresses from an undifferentiated state to a differentiated one [79]. In this framework, a stem cell—represented by a ball—rolls down the rugged landscape and eventually enters one of the valleys at the foot of the hill (Figure 1a). These valleys are the attaractors of a system [80]. Systems with more than one attractor are called “multistable” and have been experimentally observed in other biological contexts as well, such as during development, where one progenitor cell can give rise to two or more differentiated cell fates [81]. These attractors are governed by the complex, interlinked EMT regulatory networks operating at multiple levels—transcriptional, translational, post-translational, and epigenetic [80,82] (Figure 1a). The presence of these attractors raises the possibility that isogenic cells can respond differently to the same dose and duration of identical EMT-inducing stimuli. This cell-to-cell variability can arise due to multiple factors including cell cycle stage, stochasticity/fluctuations in biochemical reaction rates, concentrations of various molecular species, etc. [83]. Indeed, NMuMG mammary epithelial cells exposed to specific durations and concentrations of TGF-β were observed to respond largely in a bimodal manner—one subpopulation readily lost E-cadherin expression while the other remained epithelial; a similar trend was observed consistently across a larger panel of cell lines [47]. Notably, this bimodality existed only at intermediate concentrations or durations of TGF-β treatment; all cells maintained an E-cadherin^high^ state at very low concentrations, and all of them switched to E-cadherin^low^ at very high concentrations (Figure 1b). Such dose-/time-dependent bimodality indicates that isogenic cells can attain more than one phenotype under the same experimental conditions. The phenotype attained by an individual cell depends on its genetic and epigenetic background, which determines how “poised” a cell is to alter its biophysical and/or biochemical traits in response to varying extents of stimuli capable of eliciting an EMT response [32].

Multistability, or the presence of multiple attractors, can also drive non-genetic heterogeneity during chemotherapeutic responses and lead to resistance, a feature associated with EMT [28]. For instance, the treatment of a clonal cell population with apoptosis-inducing stimulus TRAIL (TNF-related apoptosis-inducing ligand) for the same duration and dose was shown to negatively affect viability only in a fraction of cells, while the rest survived [84]. This heterogeneity was attributed to a high variance in protein levels for a common set of apoptotic regulators. Such variability may contribute to treatment failure and provide a long-standing reservoir of cells that can gain drug resistance by virtue of newly acquired genetic alterations [85,86]. With this increased appreciation of the complexity associated with EMT/MET processes, we should practice caution in defining the exact parameters that should be referred to as EMT/MET (or various shades of these transitions) in vitro and in vivo to minimize further ambiguity.

The presence of multiple attractors in a given system allows another interesting dynamic property: Cells exhibiting a particular phenotype (E, M, or hybrid E/M) can transition spontaneously to another phenotype under the influence of “intrinsic noise” or “extrinsic noise” in biological systems [87]. Such “spontaneous switching” between E and M states was recently demonstrated in mouse prostate cancer cells: The cell population was first sorted based on EpCAM and vimentin levels through fluorescence-activated cell sorting, and then cultured independently. Cells of each of the three sub-populations (EpCAM^+^ Vim^−^, EpCAM^+^ Vim^+^, and EpCAM^−^ Vim^+^), when cultured independently, were able to switch to the other two subpopulations [75]. Similar observations were made in a PMC42-LA breast cancer cell line where EpCAM levels were used to segregate cells as epithelial (EpCAM^+^) or mesenchymal (EpCAM^−^) [73]. These subpopulations underwent phenotypic transitions and reverted to the phenotypic distribution seen in the parental population. The authors demonstrated that these transitions were not driven by chromosomal instability, thus emphasizing a non-genetic mechanism underlying these phenotypic transitions. In vivo evidence for “spontaneous induction” of EMT was also reported recently in MMTV-PyMT mice [88]. However, the quantification of transition rates among different phenotypes has yet to be done rigorously. Mathematical models can play a crucial role in identifying the underlying context-dependent cues that can give rise to various EMT population distributions [89]. Future studies integrating experimental and theoretical approaches, similar to the attempts made for CSC dynamics, may pave the path to a holistic comprehension of these processes [12]. 

## 3. Hysteresis/Cellular Memory Effects during EMT Dynamics

Another hallmark of multistable systems is the possibility of cellular memory or hysteresis (Figure 1c). As discussed earlier, isogenic cells exposed to the same strength and duration of a signal may respond differently because they are placed in different attractors. Therefore, the response of a cell not only depends on the stimuli received in real time, but also on the history of input stimuli encountered previously that may have driven them to occupy specific attractors [90]. This property is typically described as “cellular memory”. One of the first reports connecting multistability to cellular memory in mammalian systems exposed HL60 cells to increasing concentrations of DMSO for 7 days to differentiate them into neutrophils (forward reaction), and subsequently these fully differentiated neutrophils were resuspended in decreasing concentrations of DMSO for the same duration (backward reaction). Interestingly, the fraction of cells expressing CD11b—the surface marker for neutrophils—was different in the two trajectories for the same concentration of DMSO treatment. This asymmetry in response was attributed to underlying multistability: Because every cell had multiple possible attractors—CD11b^hi^ and CD11b^lo^—their likelihood of acquiring one phenotype or the other depended not only on the DMSO received instantaneously, but also on all DMSO treatments received in the past [90]. Similar observations were recently made in cells undergoing EMT and their reverse MET [47,53]. HCC827 lung cancer cells treated with increasing concentrations of TGF-β to induce EMT (forward reaction) followed by progressively decreasing concentrations of TGF-β to induce MET (backward reaction) exhibited assymmetric transition trajectories, as measured by 28 markers at a single-cell level. Furthermore, some cells did not revert to the epithelial phenotype even when TGF-β was completely withdrawn, indicating cellular memory [53]. The irreversibility of EMT has also been reported elsewhere [91,92,93], most likely due to “extreme” EMT induction. Nonetheless, the mechanisms of such irreversibility have yet to be identified comprehensively. However, preliminary evidence suggests that epigenetic treatments may help disrupt such irreversibility and permit the to reversion of cells to an epithelial phenotype [94,95], as many canonical epithelial genes such as E-cadherin can be epigenetically silenced during EMT progression [96,97]. 

Compared to EMT, molecular mechanisms mediating MET are relatively less characterized [98]. GRHL2—a transcription factor that activates CDH1 (E-cadherin) and CLDN4 (Claudin-4)—and OVOL1/2 can repress EMT-associated transcription factors and drive MET [99,100,101]. However, the overexpression of OVOL2, GRHL2, or E-cadherin may not always be sufficient to drive complete MET [95,102,103,104]. These observations reinforce the aspect that cells may navigate through different paths in the multi-dimensional landscape of EMP to undergo EMT or MET in a context-dependent manner; thus, the dynamics of EMT and MET need not be always symmetrical.

The bidirectional communication between computational and experimental approaches has been pivotal in gaining new insights into the dynamics of carcinoma EMT and MET. These insights have been suggestive of potential therapeutic strategies, particularly for reducing metastatic aggressiveness that exhibits a greater dependency on cellular plasticity than genetic mutations [105]. Firstly, driving tumor cells into a “locked” or “irreversible” mesenchymal state may compromise their ability to colonize distant organs, as observed in previous reports [8,20,46,106]. Secondly, mutually inhibitory feedback loops have been identified as regulators of multiple facets of cellular plasticity in cancer progression—EMT/MET [17,107], mesenchymal–amoeboid transition (MAT), and amoeboid–mesenchymal transition (AMT) [108], matrix-detached and matrix-attached states [109], and metabolic switching between oxidative phosphorylation and glycolysis [110]. Congruently, such feedback loops have also been observed to mediate various cell-fate decisions during embryonic development [111]. Disruption of such feedback loops may reduce cellular plasticity and curb metastatic potential in vivo [47]. Finally, the mechanisms responsible for maintaining the hybrid E/M phenotype(s)—considered more aggressive and metastatic in contrast to “extremely epithelial” or “extremely mesenchymal” ones [20,112]—can be targeted to reduce metastasis. These hybrid E/M cells exhibit higher tumor-initiating or cancer-stem-cell-like (CSC-like) properties than extremely epithelial or extremely mesenchymal populations [13,20], a notion supported by accumulating clinical evidence wherein co-expression of epithelial and mesenchymal markers tends to be associated with a poor patient survival across cancer types [71].

While an iterative interplay between mathematical models and experimental data has unraveled key design principles of the dynamics of cellular plasticity and heterogeneity during EMT/MET, many open questions remain. For instance, it remains to be identified how many hybrid E/M phenotypes exist and what the similarities and differences in their functional attributes are. While mathematical models of different regulatory networks have a common prediction that EMT/MET is not a binary process, different numbers of hybrid E/M states with varying molecular signatures have been predicted [113,114,115]. Which combination of molecular markers is most appropriate to experimentally identify these hybrid E/M phenotype(s) needs to be commonly agreed upon [71]. A robust identification of such markers could help affirm/falsify the predictions from these models, and fuel this interdisciplinary approach to classification of the ‘”common organizing principles” underlying the “myriad phenotypic complexities” [116] associated with various aspects of tumor progression, including metastasis.

## 4. Phenotypic Interconversions of Cancer Stem Cell Populations

**Figure 2:**
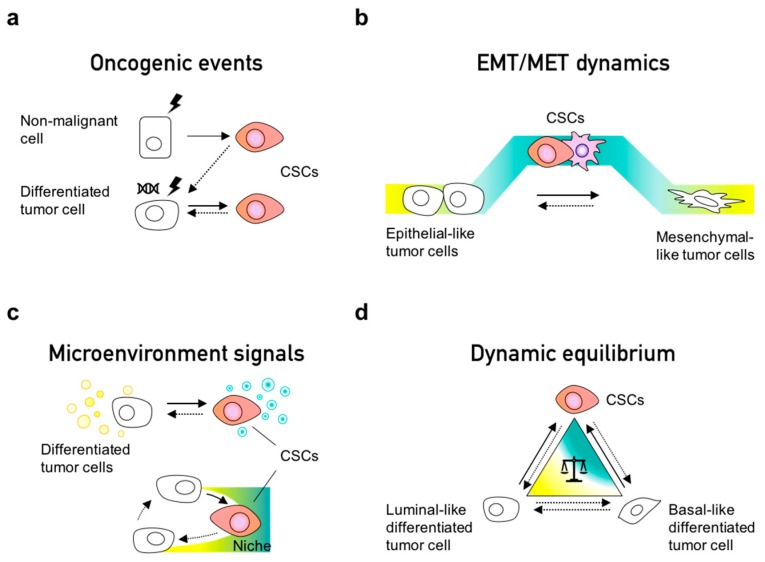
Origins and dynamics of cancer stem cells (CSCs). (**a**) CSCs can originate from normal cells during malignant transformation, induced by oncogenic events. Separately, additional genotoxic insults on malignant cells can lead to a dedifferentiation process of differentiated tumor cells into CSCs. Black and white cells are differentiated cells and colored cells are CSCs. (**b**) EMT/MET generates stem cell properties in cancer cells; however, extreme EMT can cause a loss of stemness potential. Therefore, cell plasticity and reversibility are important features in reversion to hybrid E/M states. (**c**) Microenvironmental signals can induce stemness in non-CSCs, e.g., cytokines such as IL-6 or TGF-β. In addition, tumor cells can hijack the niche of normal stem cells, inducing dedifferentiation and stemness in cancer cells. (**d**) Tumor cell populations tend to maintain their inherent proportion of CSCs. Differentiated phenotypes and lineages in tumours, either luminal and basal, can switch to CSCs when these are depleted or diminished due to experimental approaches or anticancer treatments.

Cancer stem cells (CSCs) are cells with self-renewal capacity that lead tumor initiation and give rise to the differentiated cells which constitute phenotypically heterogeneous tumors [29,117,118]. The notion of their existence has been around for over a century, but it gained more attention when the first CSC-specific markers were identified in hematological and solid tumors [119,120,121]. These populations have been reported to originate from normal stem cells, progenitor cells and differentiated cells that undergo a dedifferentiation process during malignant transformation (Figure 2a). Several markers have been described to define CSC populations in different cancer types; for instance, CD24^−/low^/CD44^high^ markers delineate a common CSC population for breast cancer, colorectal cancer, ovarian cancer, liver cancer, and others [122]. Interestingly, this population is characterized as the mesenchymal-like CSC population in breast cancer [123]. ALDH (aldehyde dehydrogenase) activity is another pan-CSC marker which can be employed for dissecting epithelial-like or E/M-hybrid-like CSCs [123], suggesting the existence of different CSC subsets within the same tumor depending on their EMT state. Indeed, CSCs can also exist in a quiescent or highly proliferative state, as has been reported since early seminal studies [124]. 

The CSC phenotype is a dynamic state rather than a fixed population, as confirmed by lineage tracing in breast cancer models [125] and in human colorectal xenotransplants [30], wherein a continuous turnover of CSCs has been observed. Other in vitro models have also shown that CSCs can arise from non-CSCs [12,126,127]; for instance, cells undergoing EMT can convert from non-CSCs to CSCs [22] (Figure 2b). A recent study using lineage tracing and RNA-seq demonstrated that EMT occurs continuously during early tumorigenesis in individual clones [128], thus enabling CSC properties. EMT implies a transdifferentiation from an epithelial to a mesenchymal phenotype; therefore, it is not surprising that cells first dedifferentiate—increasing stemness—prior to their entry into the mesenchymal-like state. Thus, consistent with in silico predictions from mechanistic mathematical models [21], stemness has been observed to peak in the hybrid E/M state(s) rather than terminal epithelial or mesenchymal states [20,46]. Interestingly, in breast cancer, non-CSC to CSC conversions have been observed to occur more often in the basal-like subtype than in the luminal-like subtypes. This difference is due to the maintenance of bivalent or “poised” chromatin marks on the ZEB1promoter—an important EMT inducer—able to quickly respond to environmental signals [129]. Indeed, such poised marks have also been demonstrated for crucial cell-fate regulators in the differentiation of embryonic stem cells [130]. 

Dynamic reversible processes such as EMT can mediate interconversion among CSCs and non-CSCs. Besides EMT, cancer cells can also take alternate routes to acquire CSC properties, which include undergoing a dedifferentiation process by oncogenic transformation [127,131], acquisition of new mutations [132,133], reversible senescence [134], and in response to inflammatory signals from the microenvironment [11] (Figure 2a–c). In colorectal differentiated tumor cells, NF-κB signaling has been shown to activate the Wnt pathway to induce dedifferentiation, re-expression of Lgr5, gain of stem cell properties, and increased tumor initiation ability [135]. In addition, cancer cells can outcompete resident stem cells and occupy their supportive niches to acquire stem cell properties [11,136,137]. Interestingly, the depletion of Lgr5^+^ cells ceases tumor growth of CRC, yet tumor growth is restored by the spontaneous reappearance of Lgr5^+^ cells in a dedifferentiation event in the primary tumor but not in the metastatic liver site, suggesting the absence of a CSC-supportive niche in the liver [30,138]. Overall, these studies indicate that the CSC state is a dynamic and plastic condition coordinated by tumor intrinsic and extrinsic processes.

Phenotypic plasticity can explain the continuous appearance of CSCs reported in clonal evolution studies [125,139]. In fact, not all cancer types follow the hierarchical CSC model, as reported in melanoma and pancreatic studies by the lack of clonal expansion [139,140,141,142]. This observation can be a consequence of highly plastic tumors that continuously interconvert CSC states in equilibrium. In pancreatic cancer, CD133^+^ tumor-initiating cells are transiently and continouously generated, since their presence is required for tumor generation [139]. Therefore, the CSC phenotype—transient or sustained—seems to be crucial for tumor and metastasis initiation.

## 5. Dynamic Equilibrium within Cancer Cell Populations

Some studies have demonstrated a dynamic equilibrium between CSC and non-CSC populations (Figure 2d) [143,144]. Similarly to complex systems, tumors can maintain a phenotypic equilibrium for functional redundancy and feedback control [145]. A pioneering study demonstrated how a mixed population of CD44^high^ and CD44^low^ cells sorted from HME (normal human mammary epithelial cells) restored the parental stem-cell-like population. CD44^high^ cells were observed to undergo differentiation while the CD44^low^ population transitioned into the stem-cell-like CD44^high^ phenotype, implying the existence of homeostatic control at population level. An alternate explanation could be different growth rates among stem-cell-like and differentiated cells; further investigation is required to deconvolute these different hypotheses [127]. Another landmark study combined the use of mathematical models and experimental approaches to characterize the equilibria of CSC and non-CSC populations [12]. Two breast cancer cell cell lines (SUM149 and SUM159) used in this study comprised different distributons in terms of luminal-like (L), basal-like (B), and stem cell-like (S) subpopulations. When these three subpopulations were segregated and cultured separately, all subpopulations returned to the original equilibrium of the parental cell line (SUM149 or SUM159, respectively), reminiscent of observations made in PMC42-LA systems [73]. Thus, de novo CSCs emerged independent of the starting point—L or B cells. These findings were later explained using a mathematical model proposing that phenotypic distributions in a given population (cell line) can be maintained due to stochastic cell-state transitions [12]. Another study using these cell lines showed how the aberrant regulation of cell fate determinants such as Slug can alter the balance of interconversion between luminal and basal cell populations [126]. Therefore, a perturbation in key regulatory genes can alter the relative stability of various possible attractors, and consequently generate different phenotypic distributions [146,147]. 

The population dynamics of cancer cells can also be influenced by extrinsic input fluctuations from microenvironmental signals. For instance, Zeb1 is epigenetically controlled, with bivalent histone marks allowing quick responses to TGF-β signals, impacting the dynamic equilibrium among CD44^low^-non-CSCs and CD44^high^-CSCs [129]. Thus, epigenetic marks can directly govern cell state transitions by affecting the transcriptional accesbility of genes involved in cellular plasticity [148]. TGF-β signaling also participates in maintenance of the equilibrium of non-CSC and CD133^+^ CSCs, as reported in breast and colon cancer cells in vitro [144]. It is of note that TGF-β signaling also modulates the dynamic heterogeneity in embryonic stem cells by altering the balance of Nodal and BMP pathways [149]. In breast and prostate cancer, inflammatory cytokines such as IL-6, which are also involved in EMT [150], establish a dynamic balance of CSCs and non-CSCs. IL-6 secretion maintains the balance of newly generated CSCs and the CSC differentiation to non-CSCs [131]. In agreement with these studies, stochastic simulations estimated the rates of interconversion between epithelial-proliferative and mesenchymal-quiescence states in breast CSCs. Similarly, disrupting the inflammatory feedback loop signals of IL-6, Stat3, and NF-κB has been predicted to serve as a therapeutic intervention able to eliminate both types of CSCs [151]. This model prediction has yet to be experimentally tested.

## 6. Non-Cell Autonomous Effects of the EMT Process and CSC Identity

Tumors have been postulated to display collective behavior and can be viewed as a community of social cells [78,152]. Indeed, swarm-like behavior has been proposed to facilitate optimal utility of tissue space and induce motility beyond a threshold of tumor population density [153]. This collective behavior could be the result of the synchronized EMT evident in migrating individual mesenchymal cells documented in developmental and cancer models [35,88,154]. Synchronized EMT in cell populations can be observed in embryonic cells that ingress and form the mesoderm in the invagination and epiboly steps of gastrulation. The origins of this spatiotemporal synchrony are often assigned to the “organizer” group of cells, such as the Nieuwkoop center and Spemann organizer, which demarcate the onset of EMT in *Xenopus* embryos [155]. The signal gradients emanating from these node organizers, Wnt/β-catenin, and Nodal/TGF-β dictate the space and time of EMT during gastrulation [155,156]. In cancer, such structures have not yet been determined, as EMT is not likely to be restricted to a particular time or space; instead, it can occur spontaneously during different stages of disease progression and depending on microenvironmental changes. 

The current observations of EMT in cancer have been mainly based on detecting morphologically visible invasive cells at tumor margins. Recent evidence suggests continuous EMT in the early stages of tumor development in different clones [128], even in preneoplastic stages [157]. Tumor marginal invasion has been captured by intravital microscopy (IVM), showing the occurrence of spontaneous EMT in individual cells of MMTV-PyMT breast tumors [88,158]. Another intravital imaging study implicated TGF-β in coordinating the local switch from attached groups of cells to cells displaying individual motility [159]. Overall, EMT might be synchronized at a population level in cancer. 

Interestingly, E-cadherin has been reported to function as a sensor of cell population density, providing a mechanism by which cell populations may reach phenotypic equilibria through EMT in tissues. Mechanistically, E-cadherin can sense low cell densities and increase the availability of growth receptors, thus favoring downstream EGFR/ERK signaling and β-catenin stabilization to stimulate growth [160,161,162]. Computational studies have modeled “anti-social” behavior of E-cadherin-negative cells, typical of EMT-like cells, and predicted that their presence could disrupt existing population dynamics, depending on external environmental factors such as calcium levels [160]. This instance is a good example of “secrete-and-sense cells”, by which an EMT event could alter homeostasis and influence the entire population. 

## 7. Spatiotemporal Dynamics of EMT and CSCs

Cellular phenotypes displaying varying levels of EMT and/or CSCs have been witnessed in vitro and in vivo; one recent focus has been the identification of their spatial localizations within a tumor. One of the first reports on spatial heterogeneity in EMT proved a higher nuclear localization of β-catenin at the invasive edge of primary colorectal carcinomas, while a more cytoplasmic and membranous staining was evident in central tumor areas [163]. Concurrently, membranous E-cadherin was largely retained in central tumor areas but lost at the invasive edge [164,165]. More recently, subsets of CSCs (CD44^+^/CD24^−^ and ALDH^+^) with varying EMT status (mesenchymal and hybrid E/M, respectively) have been described in breast cancers [123,166], with the mesenchymal subset located at the invasive edge and the hybrid E/M subset located in the tumor interior. This spatial distribution can be attributed to gradients of EMT-inducing signals and cell-to-cell communication in tumors [167]. In both aforementioned cases, the mesenchymal subpopulation at the invasive edge of primary tumors has been reported to be quiescent, while the central tumor subpopulation tends to be proliferative [123,163], consistent with the “go-or-grow” (i.e., migrating cells have low proliferation rates) paradigm, as witnessed in in vitro analysis of EMT and cell cycle regulators [26,168]. Single-cell transcriptomic analysis of primary head and neck tumors has further strengthened the finding of prominent mesenchymal features at the invasive edge [48]. Thus, a primary tumor may contain spatially distributed cells with varying extents of EMT [68,167].

Spatiotemporal patterns of EMT and non-EMT cells have been observed in vitro as well. EMT-like cells can induce EMT across the population by paracrine and/or juxtacrine signaling and generate an equilibrium of EMT-induced and non-EMT cells in tumor cell clusters [47,68]. The processes by which a cell population reaches these equilibria in a spatiotemporal manner require further investigation, and this is another example where mathematical modeling could reveal the underlying mechanisms. 

This spectrum of heterogeneity has also been observed beyond the primary tumor in disseminated circulating tumor cells (CTCs) from patients across cancer types [59,69,169,170]. CTCs can migrate either as individual cells or in units of two or more cell clusters [169]. Various spatiotemporal patterns in EMT phenotypes may influence frequencies and size distributions of CTC clusters [171], which are considered the primary harbingers of metastasis [172]; thus, an understanding of their characteristics, such as size distribution, frequency, ability to traverse capillaries [173], and molecular profiles of their tumor and/or stromal cell populations [174], holds promise in highlighting new therapeutic vulnerabilities. Connecting these traits of CTC clusters to spatiotemporal dynamics of EMT in a primary tumor has yet to be undertaken comprehensively. Since these CTC clusters can contain various non-cancerous cells such as platelets and fibroblasts, their presence may have many functional consequences in accelerating metastasis; for instance, macrophages may facilitate transendothelial migration and neutrophils may drive cell cycle progression during circulation [175,176]. Thus, future efforts should focus on the mechanistic underpinnings of various modes of cell-to-cell communication, coordination, and cooperation among tumors and stromal cells during the various steps of the metastasis–invasion cascade.

## 8. Conclusions

Dynamic cell plasticity increases the phenotypic heterogeneity of tumors and thus tumor versatility at the population level. This phenomenon increases the complexity of the mechanisms underlying carcinogenesis, metastasis, and its treatment. The study of non-static systems is technically challenging, but the emergence of new techniques able to study single cell phenotypes and cell state transitions through reporter cell lines, real-time imaging in combination with mathematical modeling, and big data analysis sheds light on the existence of dynamic behaviors. Future studies need to focus on decoding the molecular mechanisms responsible for such emergent behaviors at cellular and population levels. An individual renegade cell has long been considered to be the unit of cancer progression. However, with accumulating evidence about collective phenomena at a tissue level, such as engineering of the primary tumor and/or metastatic niche [136,177], collective migration [175], and metabolic synergy [178], we must focus on non-cell autonomous mechanisms of cellular plasticity in the tumor microenvironment [179]. In addition, new studies should attempt to elucidate the nonlinear dynamics of cell-to-cell communication and co-operation in tumor progression. Such an integrative and dynamic understanding will steer us towards outsmarting cancer through innovative approaches such as blocking cellular plasticity bidirectionally and designing adaptive therapies that take into account the evolution of resistance [180].
